# Insights from a dataset on web system adoption among small and medium-sized enterprises (SMEs): A survey in Malaysia

**DOI:** 10.1016/j.dib.2026.112834

**Published:** 2026-05-10

**Authors:** Mathivannan Jaganathan, Subramaniam Kolandan, Logeswari Uthama Puthran, Oussama Saoula

**Affiliations:** a School of Business Management, College of Business, Universiti Utara Malaysia, Sintok, Kedah, Malaysia; b Department of Business Management and Entrepreneurship, Faculty of Management and Economic, Universiti Pendidikan Sultan Idris, Malaysia; c Institut Aminuddin Baki, Ministry of Education Malaysia, Malaysia

**Keywords:** Web system adoption, Relative advantage, Compatibility, Top management support, Security, Cost

## Abstract

This dataset comprises survey responses from 207 actively operating small and medium-sized enterprises (SMEs) in Malaysia, collected to examine factors influencing the adoption of web systems among SMEs. The data include measures of relative advantage, compatibility, security, cost, top management support, and web system adoption, captured using validated multi-item Likert scales. Data were collected via an online survey. The dataset is structured into demographic profiles and item-level responses for all constructs, enabling researchers to perform descriptive, correlational, and predictive modelling analyses. The dataset is openly accessible through Mendeley Data and can be reused for comparative studies, meta-analyses, replication work, or methodological demonstrations involving technology adoption among SMEs. The study provides valuable insights for managers and practitioners seeking to revolutionise technology adoption initiatives, particularly in SMEs, to improve day-to-day business operations. Furthermore, the dataset is relevant to policymakers seeking to promote technology adoption among SMEs in Malaysia.

Specifications Table

**Table:** 

Subject	Management of Technology and Innovation and Entrepreneurship
Specific subject area	Technology adoption
Type of data	Survey data, tables, and figures
Data collection	The dataset was analysed by collecting raw data through an online form questionnaire. Major variables adopted from past studies. The data was analysed by using SmartPLS 4 for the Structural Equation Model (SEM) while SPSS 26 was used to analyse descriptive data. Valid answers were obtained from 207 Small and Medium-sized companies in Malaysia. The validity and reliability of the measures were verified.
Data source location	*Country: Malaysia*
Data accessibility	Repository name: Data and the questionnaire used in the survey are available in this article. https://data.mendeley.com/datasets/r5kvc88wgk/2
Related research article	*None*

## 1 Value of the Data

• This data set provides item-level survey responses on web system adoption, focusing on the organisational and technological factors among Malaysian SMEs and promoting secondary analysis without reliance on aggregated scores.
• The data can be reused in future research to conduct descriptive statistics, exploratory analysis, correlation analysis, and also used as an alternative modelling approach by employing different statistical techniques or theoretical frameworks.
• Policymakers and practitioners may use the dataset as descriptive evidence to understand patterns of web system usage among SMEs in Malaysia, without reliance on inferential claims.
• The constructs included in this study are widely used in technology adoption research, making them highly transferable to other cultural, industrial, and organisational contexts and can also be reused to conduct cross-country comparisons, sector-specific analyses, or longitudinal extensions in future studies.
• This dataset is beneficial for meta-analyses and systematic reviews, especially those synthesising determinants of technology adoption in SMEs, due to item-level measurements rather than only aggregated scores.

## 2 Background

This dataset is grounded in the theoretical foundations of the technology–organisation–environment framework. This dataset adopts the TOE framework to explore the process through which technological, organisational, and environmental contexts act upon adopting and implementing technological innovations. Based on the Technology–Organisation–Environment (TOE) framework, the dataset in this study captured key technological and organisational variables that regularly shape technology adoption decisions in Malaysian SMEs, including relative advantage, compatibility, security, cost, top management support, and web system adoption. These well-researched constructs were selected because they are widely used in technology adoption research. Additionally, these constructs facilitate comparisons and integrations with studies conducted in other countries or business contexts.

The dataset was structured through a questionnaire to target SME owner-managers with direct knowledge of their firms’ operational processes and digital practices. The survey instrument includes item-level measures for each construct, as well as demographic information and firm characteristics. The dataset empirically tested the hypothesis that relative advantage, compatibility, cost, and security influence the adoption of the relationship web system, incorporating top management support as a mediating variable. The purpose of this dataset is to provide a high-quality digital adoption dataset at the SME level, which can be accessible to researchers, policymakers, and practitioners.

## 3 Data Description

The facts and data presented in this paper were collected from small and medium-sized enterprises through a well-structured online survey. The online questionnaire was distributed via email. The questionnaire was divided into two sections, Sections A and B. Section A included questionnaire items related to research variables (web system adoption, top management support, relative advantage, security, compatibility, and cost). In contrast, Section B was used to collect demographic information (age, gender, education, and SME sector). 5-point Likert scales were employed to measure dependent variables (web system adoption), independent variables (relative advantage security, compatibility and cost) and mediating variables (top management support). In addition, the survey incorporates an 11-item scale for web system adoption [[1], [2]], 6-items for top management support [3], 4-items scale for compatibility [4], 6-item for relative advantage [[5], [6]], 6-items for security [4] and 4-items for cost [7] (Refer to Table 1)

**Table 1:** Survey instrument.

Constructs	Items	Scale	Source
Relative Advantage	RA1 Web system allow better communication with our customers. RA2 Web system increase the profitability of our organisation. RA3 Web system create an electronic presence for our brands. RA4 Web system reduce costs (e.g., communication, advertising, marketing, travel, and R&D). RA5 Web system allow us to enter new businesses or markets. RA6 Web system improve our web presence.	1–5 Likert	[[5], [6]]
Security	SEC1 Our firm does not have confidence in the online payment system. SEC2 Our firm is concerned that information involved in a transaction over the internet is not secured. SEC3 Our firm lacks confidence on the security of internet transactions. 1 2 3 4 5 SEC4 Our firm lacks confidence in using a credit card to make payment through Internet. SEC5 Our firm worried that our business transactions on the Internet can be read and seen by others. SEC6 Our firm concerned about the security of data and transactions over the Internet.	1–5 Likert	[4]
Compatibility	COMP1 The use of web system fits the work style of the firm. COMP2 The use of web system is fully compatible with current business operations. COMP3 Using web system is compatible with your firm's corporate culture and value system. COMP4 The use of web system compatible with existing hardware and software in the firm.	1–5 Likert	[4]
Cost	COST1 The costs involved in the adoption of web system would be far greater than the expected benefits. COST2 The cost of maintaining web system tools would be very high for our enterprises. COST3 The cost involved in providing support systems for web system would be too high. COST4 The amount of money invested in training employees to web system would be very high.	1–5 Likert	[7]
Top Management	TMS1 Top management actively seeks middle managers’ opinions and ideas on issues related to web system adoption. TMS2 Top management is open to new ideas and initiatives from team members regarding web system adoption. TMS3 Top management appreciates middle managers' experiments with new ideas and approaches for web system adoption. TMS4 Top management appreciates middle managers' experiments with new web systems. TMS5 Top management ensures that the interests of middle managers are considered when making strategic decisions about web system adoption. TMS6 The organisation establishes a committee at various levels to measure the commitment of top management towards web system adoption.	1–5 Likert	[3]
Web System Adoption	WS1 Our firm uses internet websites to find information relevant to our firm businesses. WS2 Our firm uses Internet websites to find information about other business stakeholders WS3 Our firm uses the firm’s website for presenting the firm's profile, product and service information. WS4 Our firm uses the firm’s website for exchanging information with customers online e.g. discussion forums. WS5 Our firm uses the website for customers to place orders online. WS6 Our firm uses the website for suppliers to view suppliers’ stock quantity in the firm. WS7 Our firm places orders online to suppliers WS8 Our firm fully getting orders online from customers. WS9 Our firm fully getting orders online from suppliers. WS10 Our firm uses the firm’s website for receiving customers' complaints about the firm products and services offered. WS11 Our firm uses the firm’s website for updating customers on the new products and services offered.	1–5 Likert	[[1], [2]]

To determine the appropriate sample size for this study, a priori power analysis was conducted using G*Power 3.1.9.7 software with five predictor variables: relative advantage, compatibility, security, cost, and top management support. Effect size (f²): 0.15 (medium effect size), alpha level (α): 0.05, power (1-β): 0.95 and 5 predictors were set as a parameter for the power analysis [8]. Based on these parameters, the power analysis indicated that a minimum sample size of 138 participants is required to detect a medium effect size with 95% power at a 5% significance level. This sample size guarantees that the study has sufficient power to detect significant correlations between the predictor factors and the dependent variable. [9]. However, this study used 207 complete responses from an online survey of small and medium-sized enterprises (SMEs) in Malaysia, exceeding the minimum sample size.

In studies of small and medium-sized enterprises (SMEs) in developing countries such as Malaysia, it is often necessary to distribute a larger number of questionnaires to participants due to low response rates, a method frequently used in social science research. Therefore, this study determined that the number of questionnaires distributed should be increased to 5 times the required sample size, resulting in a total of 690 questionnaires. The survey link was sent to the email addresses of the selected companies. Respondents were given two weeks to complete the survey, followed by a reminder after two weeks; if no response was received within a further two weeks, respondents were considered non-respondents. This study achieved a moderate response rate of 31.16% via an online survey. Six hundred ninety questionnaires were distributed to entrepreneurs/owner-managers of SMEs in Malaysia. A total of 215 questionnaires were returned. After excluding eight incomplete responses, 207 questionnaires were deemed usable for the final analysis.

Table 2 displays the demographic information of SME entrepreneurs/owner-managers who have used web systems. It also presents the frequency distribution of respondents based on 207 usable returned questionnaires.

**Table 2:** Demographic of participant (N = 207).

Demographic Variables	Category	Frequency	Percentage %
Gender	Male	178	86
	Female	29	14
Age	Below 30	45	21.7
	31–40	78	37.7
	41–50	62	30.0
	51–60	8	3.9
	61 and above	14	6.8
Race	Malay	117	56.5
	Chinese	61	29.5
	Indian	27	13
	Others	2	1.0
Education	Masters	8	3.9
	Undergraduate	93	44.9
	Diploma	66	31.9
	Certificate	40	19.3

Table 2 presents the frequency distribution of respondents based on 207 returned questionnaires. Table 2 shows that most respondents are male, with 178 (86%) and just 29 females (14%). Regarding age, 78 (37.7%) entrepreneur/owner-managers are within the age bracket of 31–40, 45 (21.7%) are below 30 years of age, 62 (30.0%) are within 41–50 years of age, and only 8 (3.9%) entrepreneur/owner-managers fall into the category of 61 and above age group. The dataset also found that the majority of respondents (117, 56.5%) were Malay, followed by Chinese (61, 29.5%), Indians (27, 13%), and others (2, 1.0%). Regarding respondents’ academic qualifications, Table 2 shows that the majority (44.9%) hold first degrees, while 8 (3.9%) hold master's degrees.

Table 3 presents the composite reliability, average variance extracted (AVE), and indicator loadings for the independent variables (relative advantage, compatibility, security, cost), the mediator (top management support), and the dependent variable (web system adoption). The composite reliability values of the four reflective latent constructs exceeded the recommended cut-off value of 0.7, ranging from 0.82 to 0.93. Besides, convergent validity results are also greater than the acceptable threshold of 0.5 [10]. As shown in Table 3, AVE values are in the range of 0.53 and 0.57. Based on this criterion, the square root of the AVE of a construct should be higher than the correlation between the constructs and all other variables, or the square root of AVE on the diagonal should be higher than the correlation on the off-diagonal.

**Table 3:** Results summary for reliability, validity and outer loadings.

First Order Construct	Scale Type	Item	Loadings	AVE	Cronbach`s Alpha	Composite reliability
Relative Advantage	Reflective	RA1	0.72	0.57	0.85	0.86
		RA2	0.81			
		RA3	0.78			
		RA4	0.67			
		RA5	0.77			
		RA6	0.76			
Compatibility	Reflective	COMP1	0.72	0.56	0.77	0.87
		COMP2	0.73			
		COMP3	0.79			
		COMP4	0.75			
Security	Reflective	SEC1	0.74	0.57	0.85	0.89
		SEC2	0.65			
		SEC3	0.79			
		SEC4	0.80			
		SEC5	0.76			
		SEC6	0.76			
Cost	Reflective	COST1	0.67	0.53	0.71	0.71
		COST2	0.77			
		COST3	0.72			
		COST4	0.75			
Top Management Support	Reflective	TMS1	0.71	0.55	0.80	0.82
		TMS3	0.62			
		TMS4	0.78			
		TMS5	0.80			
		TMS6	0.71			
Web System Adoption	Reflective	WS1	0.71	0.54	0.91	0.92
		WS2	0.76			
		WS3	0.77			
		WS4	0.75			
		WS5	0.73			
		WS6	0.67			
		WS7	0.80			
		WS8	0.78			
		WS9	0.77			
		WS10	0.77			
		WS11	0.55			

Note: Items Deleted Due to Low Loadings= TMS2.

Overall, the HTMT ratios presented in Table 4 support the discriminant validity of the measured constructs. Discriminant validity ensures that constructs that are supposed to be distinct are indeed different from one another [[11], [12]]. The HTMT ratio is a stringent criterion used to evaluate this aspect in structural equation modelling, with a commonly accepted threshold of 0.90. All HTMT ratios presented in Table 4 are below the conservative threshold of 0.90, confirming that the constructs COST, COMP, RA, SEC, TMS, and WS are adequately distinct. Henseler et al. [13] also suggested a threshold value of 0.90 if constructs are conceptually very similar. Therefore, HTMT in this study demonstrates strong discriminant validity.

**Table 4:** Discriminant validity - heterotrait-monotrait ratio.

	COST	COMP	RA	SEC	TMS	WS
COST						
COMP	0.55					
RA	0.52	0.89				
SEC	0.56	0.77	0.86			
TMS	0.78	0.73	0.51	0.55		
WS	0.36	0.59	0.61	0.68	0.41	

**Note:** COST=Cost Comp=Compatibility RA=Relative Advantage SEC=Security TMS=Top Management Support WS= Web System Adoption.

**Fig. 1:**
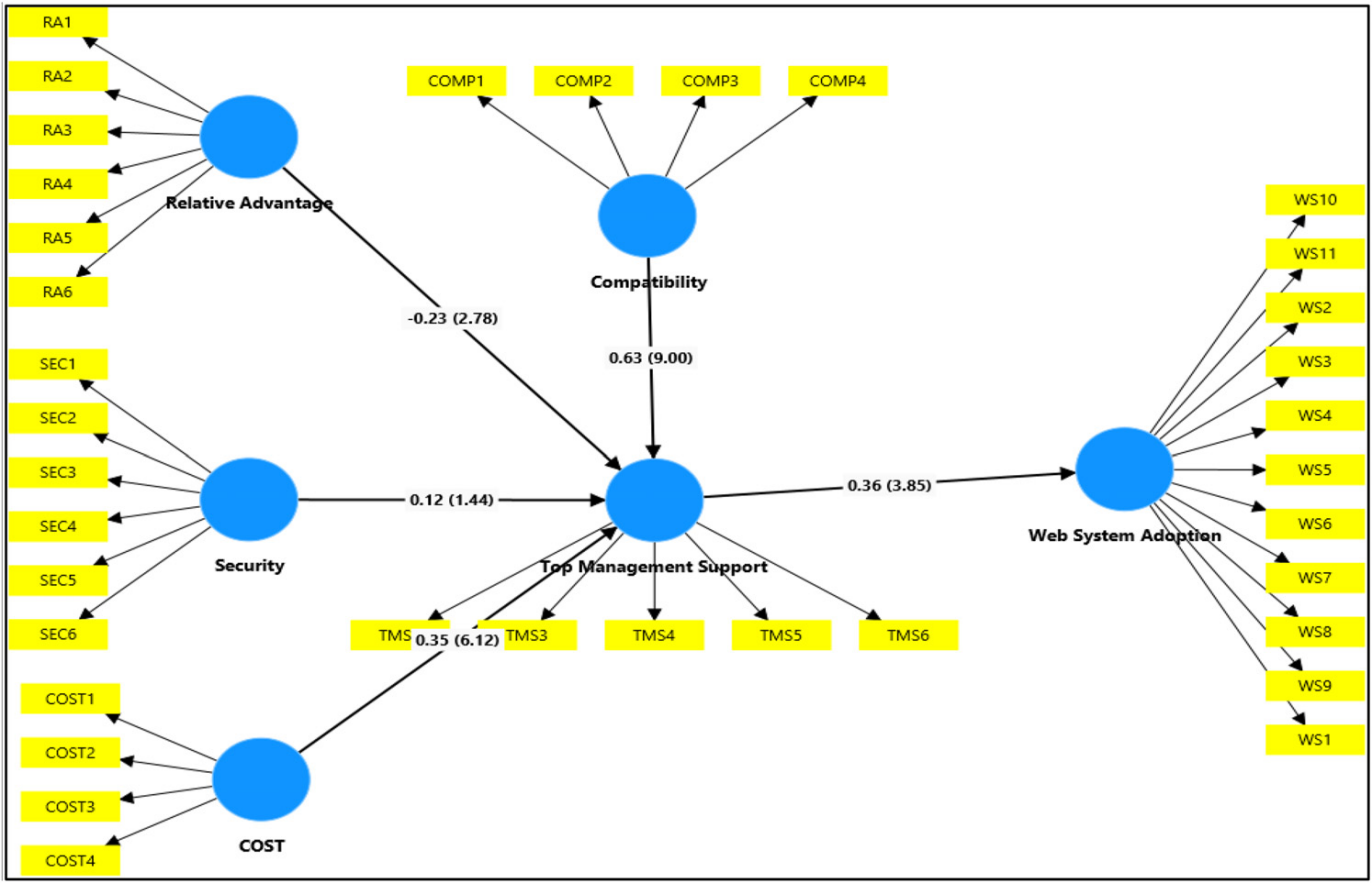
Structural model of the study. ***Note:*** COST=Cost Comp=Compatibility RA=Relative Advantage SEC=Security TMS=Top Management Support WS= Web System Adoption.

Fig. 1 presents the measurement structure of the survey dataset, illustrating the relationship between latent constructs and their corresponding item-level indicators used to operationalise web system adoption and related organisational and technological variables among SMEs.

Cohen’s effect size estimation was calculated to assess the effect size. As a rule of thumb, values higher than 0.02, 0.15 and 0.35 depict small, medium and large f2 effect sizes [[12], [13], [14]]. Therefore, the effect size was examined and reported in Table 5. The data in Table 5 show that cost and top management support have medium effect sizes, while relative advantage and security have relatively small effect sizes. On the other hand, compatibility has a large effect size.

**Table 5:** Coefficient of determination & effect size (f2).

Constructs	R^2^	Effect Size (f²)	Predictive Relevance (Q²)
Web System Adoption	0.13	-	0.15
Top Management Support	0.62	0.15	0.59
Cost	-	0.25	
Compatibility	-	0.49	
Relative Advantage	-	0.05	
Security	-	0.02	

The predictive relevance of the model is assessed through the PLSpredict procedure, as suggested by Shmueli et al. [15] The Q² is larger than 0, implying that the model has sufficient predictive relevance. Based on the PLSpredict procedure, the predictive relevance Q² values for web system adoption and top management support are 0.59 and 0.15, respectively, indicating that the model has a predictive relevance because the Q² values are above zero.

## 4 Experimental Design, Materials and Methods

The target population for this study comprises entrepreneur-owner-managers of SMEs. The population selected for this study comprises all sectors, categories, and established businesses currently operating in Malaysia and registered with the SME Corporation of Malaysia. For this study, SMEs, which are classified as micro-enterprises, were excluded because the study focuses on companies that use the system extensively in their back-end processing. In this study, SMEs were defined based on the National SME Development Council (NSDC) as reported in the SME Annual Report 2022/2023 [16].

The organisation, a small and medium-sized enterprise (SME), was purposefully selected as the unit of analysis for the purpose of this study, regardless of the industry category. For this reason, the entrepreneur/owner-manager of the organisation was selected as a key respondent. Entrepreneur/owner-managers were chosen for this study due to their ability and knowledge of the firm’s operation and administration activities. Previous researchers have enormously treated entrepreneurs/owner-managers as key respondents in past information system adoption research [17].

For this study, the probability sampling method employed was simple random sampling. This approach is renowned for its straightforwardness. With this sampling method, every object in the population has the same chance of being included in the sample. Specifically, simple random sampling was used to select SMEs operating in Malaysia. Based on data from SME Corp., there are 249,934 small and medium enterprises (excluding micro-enterprises) out of a total of 1173,601 SMEs in Malaysia.

The table was used to determine the subjects in which the researcher selected the desired number of random numbers, given the maximum and minimum values that can be selected without replacement. An empirical cross-sectional study was conducted to test the formulated hypotheses rigorously. The survey was conducted online, which offered an efficient and cost-effective way of reaching a broad audience. The survey was emailed, with each participant receiving a link to access and complete the questionnaire. Participants were invited to participate in the study with a brief introduction explaining the purpose of the research and the significance of their responses. As mentioned earlier, this research employed a standardised reminder protocol. The first reminder was sent two weeks after the initial invitation, followed by a second reminder two weeks later. This research carefully handles reminders by sending reminders by using the exact wording and sends them systematically to all non-responding SMEs. A total of 690 invitations were sent, and only 215 responses were received. After removing eight incomplete submissions based on predefined criteria, such as missing >20% of items, patterned responses, and inconsistent answers, 207 complete and usable responses were used for further analysis.

## Limitations

Considering these limitations is crucial to ensure the robustness and validity of the results derived from the data set. Firstly, this study employed a cross-sectional research design. This design was chosen based on several factors, including time and cost constraints, but it does not allow for the inference of causality. Furthermore, the results of this study may not be generalisable to a larger population as the characteristics of SMEs in Malaysia differ from those in other countries. Furthermore, this study achieved a response rate of 31.16%, which may introduce non-response bias, although it's common in online data collection. In addition, it's not possible to identify or characterise non-respondents, nor to determine whether their views differ systematically from those of respondents, which could affect the representativeness of the sample.

The dataset may be subject to sampling bias because the recruitment method primarily relies on online platforms. In addition, there was only one respondent per survey, which may introduce bias. Additionally, these data indicate potential gender bias in the sample due to its predominantly male composition. This is a common phenomenon in the SME establishment in Malaysia, where only 20.6% of SMEs are women-owned, naturally leading to a higher response rate from men. Additionally, this study provides a foundational understanding of web system adoption in the male-dominated SME sector. It underscores the need for more gender-inclusive research and can guide future efforts to achieve a more balanced representation.

Lastly, the dataset reflects the cultural and organisational characteristics of Malaysian SMEs. Specifically, managerial structure, digital readiness, and cultural norms may influence how respondents perceive technology-related constructs. As a result, the dataset will not be directly generalisable to SMEs, given differences in national and cultural contexts.

## Ethics Statement

All respondents were thoroughly informed about the content and the scope of the study before participation. Participation was entirely voluntary, and participants cannot be identified. The study was conducted in a manner that did not require Institutional Review Board (IRB) approval. All collected data was stripped of personal identifiers and anonymised to ensure participant anonymity.

The survey was distributed via email for data collection purposes only. Email addresses were not collected during the questionnaire. The online survey questionnaire was designed to disable the collection of any personal identifiers, including names, email addresses, IP addresses, and organisational identifiers. Responses were saved anonymously and analysed in aggregated form. No technical means exist to associate responses with individual participants or organisations.

## CRediT Author Statement

**Mathivannan Jaganathan:** Conceptualization, Methodology, Formal analysis, Writing - Original Draft, Writing - Review & Editing and Visualisation; **Subramaniam Kolandan:** Data curation, Resources, Funding acquisition; **Logeswari Uthama Puthran:** Writing – Reader, Review & Editing and Visualisation; **Oussama Saoula:** Writing - Review & Editing and Visualisation.

## Data Availability

DataCSV (Original data)
